# Influence of surface tension-driven network parameters on backflow strength†

**DOI:** 10.1039/c8ra09756a

**Published:** 2019-04-02

**Authors:** Yonghun Lee, Islam Seder, Sung-Jin Kim

**Affiliations:** Department of Mechanical Engineering, Konkuk University Seoul 05029 Republic of Korea yahokim@konkuk.ac.kr +82-0504-435-9201 +82-2-447-5886 +82-2-450-0517

## Abstract

Surface tension-driven flow is widely used, owing to its spontaneous motion, in microfluidic devices with single channel structures. However, when multiple channels are used, unwanted backflow often occurs. This prevents precise and sophisticated solution flow, but has been rarely characterized. We hypothesize that, with an analytical model, the parameters that influence backflow can be systematically characterized to minimize the backflow. In a microfluidic network, inlet menisci and channels are modeled as variable pressure sources and fluidic conductors, respectively. Through the model and experiment, the influence of each network element on the backflow strength is studied. Backflow strength is affected by the interplay of multiple inlet-channel elements. With the decrease (increase) of the fluidic channel conductance (inlet size), the backflow pressure of the corresponding inlet decreases. On the other hand, backflow volume reaches its peak value during the radius change of the corresponding inlet. In networks consisting of five inlet-channel elements, backflow pressure decreases with increasing step number. Our results provide the foundations for microfluidic networks driven by the Laplace pressure of inlet menisci.

## Abstract 2

This paper analyzes the effect of device elements on backflow of a surface tension-driven microfluidic device.

## Graphical abstract

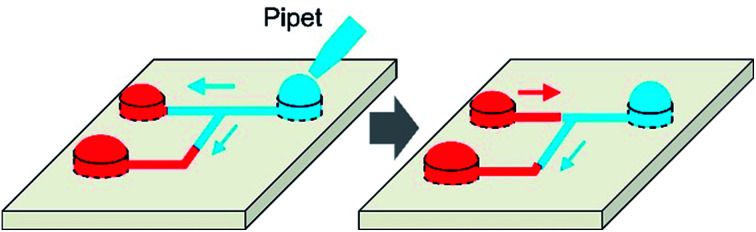

## 1. Introduction

Surface tension-driven flows are increasingly used in microfluidics, because they generate spontaneous solution flow and eliminate the need for external pumping systems. To date, studies have focused on the dynamics of surface tension-driven flows in a simple single channel.^1–7^ Such studies helped in characterizing the basic traits of the flow^8–10^ and developing surface tension-driven devices.^11–15^ Then, due to the recent demands for sequential and parallel assays, surface tension-driven devices have had increased numbers of channels and input solutions.^16–18^ Compared to flow behavior in the single channel, behavior in the multiple channels is more complex and is hard to orchestrate. However, flow characteristics in the network have been rarely studied with an analytical model.

One problem observed in a surface tension-driven microfluidic network is the backflow of liquids. Backflow can occur in the surface tension-driven devices including polymer and even paper microfluidic devices, if the devices have at least three channels with a junction and Laplace pressure difference in their inlets. For the surface tension-driven flow, inlet menisci generate inlet pressures (*i.e.*, the Laplace pressure). Importantly, this pressure significantly affects the motion of the flow. When multiple channels are prefilled with solutions and a solution is injected to an inlet, the inlet of the solution has high Laplace pressure and pushes other solutions to their corresponding inlets. The ideal case is that the initial flow direction in each channel is maintained until the flow stops. However, the flow direction in several channels can often reverse unexpectedly, resulting in backflows. Even when the filling of channels occurs by capillary action, backflow occurs. For example, when liquid fronts of two solutions merge at Y-junction and move to the junction downstream, one solution from an inlet unexpectedly moves back to the other inlet through the Y-junction.^19–22^ Such unwanted motion occurs by the imbalance of the Laplace pressure of inlets. To date, such unwanted backflows have been reported in the process of immunoassay,^18,19^ micromixing,^20^ blood typing,^21^ and cell-based assay.^22^ In the case of unexpected backflows in a surface tension-driven bioassay chip, the designed sequence of fluidic motion is changed unintentionally, and the detection of targets will fail. Thus, reducing backflow in network channels is a crucial challenge that must be overcome for such applications. Our recent study showed that backflow results from the difference between the time constant ratios of a network's inlet-channel elements.^23^ However, the contributions of network elements including channel fluidic conductance, inlet radius, and pressure to backflow strength were not analyzed in detail.

In this paper, we study the effect of each element of a surface tension-driven network on backflow strength. We model inlet menisci as variable pressure sources, and channels as fluidic conductors. Using the model, we characterize how the fluidic conductance of channels, and the radius and initial pressures of inlets, influence backflow strength. This analysis systematically explains the complex relations between network elements that affect backflow. Our model is first applied to characterize backflow in a surface tension-driven network with three inlets, and then extended to a network with five inlets.

## 2. Working process and theoretical modeling

**Fig. 1:**
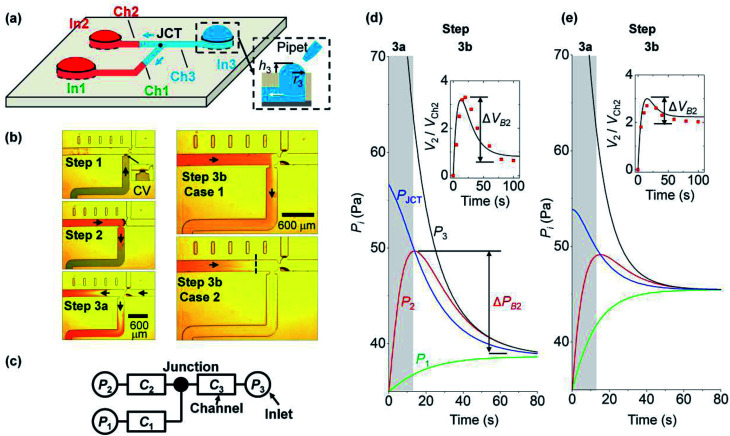
Surface tension-driven network showing backflow. (a) Generation of fluidic motion by the pressure difference of inlet menisci. Inlets (In *i*, *i* = 1 to 3) are connected to each other through channels (Ch *i*) and the junction (JCT). The inset shows the cross-section of inlet 3 with inlet radius *r*_3_ and meniscus height *h*_3_. (b) Photographs showing backflow generation process. The process consists of three steps, and backflow occurs in step 3b. Cases 1 and 2 show strong and weak backflows, respectively. In step 3, the initial pressures of inlet *i* (*P*_I*i*_) were the same for both cases with *P*_I*i*_ = 35, 35, and 100 Pa (*i* = 1 to 3). (c) Circuit diagram of the capillary network. *P*_*i*_ is the pressure of inlet *i* with radius *r*_*i*_, and *C*_*i*_ is fluidic conductance of channel *i*. (d and e) Temporal change in inlet pressures in step 3. Lines and points are the theoretical and experimental values, respectively. Inlet *i* has pressure *P*_*i*_ in step 3. Δ*P*_B2_ is the backflow pressure of inlet 2, and Δ*V*_B2_ is the normalized backflow volume. In (d), *r*_*i*_ = 2, 1, and 1 mm (*i* = 1 to 3), and *C*_*i*_ = 9, 9, and 9 (×10^−12^) m^5^ N^−1^ s^−1^. In (e), *r*_*i*_ = 1.5, 1, and 1 mm, and *C*_*i*_ = 12, 9, and 9 (×10^−12^) m^5^ N^−1^ s^−1^.

In this section, we explain the process of capillary filling and backflow generation, and then discuss the corresponding theoretical model. Backflow can occur in a channel network consisting of multiple channels, Y-junctions, and multiple branched junctions. During the merging process of the solutions at the junctions, backflows occur by the pressure difference of inlets. To describe the basic process of backflow generation, we used a network with three inlets and three channels. This is the minimum element condition that can describe the backflow in a channel network. Three steps are used to fill the network and generate the backflow. Fig. 1a depicts the fluidic motion in the network in the last step; the motion is driven by the Laplace pressure of the inlet menisci. Fig. 1b presents each step. In step 1, solution 1 (green) injected at inlet 1 stops at a capillary valve^24^ of channel 1. In step 2, solution 2 (red) injected at inlet 2 moves through channel 2 to channel 1. The fluidic motion continues until the two inlet pressures equilibrate. Backflow does not occur because only two inlet pressures are involved for fluidic motion. Step 3 consists of two parts (steps 3a and 3b). In step 3a, solution 3 (clear) injected at inlet 3 moves into channels 1 and 2. Then in step 3b, the flow direction in either channel 1 or 2 is reversed and backflow may occur. The right panels of Fig. 1b present two cases with different backflow strengths. In case 1, solution 2 exits inlet 2 and moves back from channel 2 to channel 1, resulting in the strong backflow of solution 2. If channel 1 is the target channel that each solution needs to move into sequentially, then the sequence is disrupted in step 3 by the backflow. In case 2, although the backflow of solution 2 occurs, the flow stops in channel 2 without entering channel 1, and the backflow strength is weak. Thus, the three solutions were successfully injected into channel 1 in a sequential manner.

We modeled the process of backflow generation in a surface tension-driven network (Fig. 1c). The inlet menisci were modeled as variable pressure sources because the volume of each meniscus changes in each step, which temporally varies inlet pressures. The channels were modeled as fluidic conductors. The detailed derivation process is explained in Sections 1 and 2 of ESI.† The pressure (*P*_*i*_) of inlet *i* is obtained by1
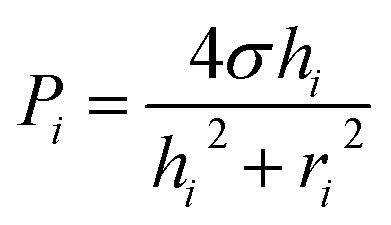
where *σ* is the surface tension of a solution injected into inlet *i*, *h*_*i*_ is the height of the convex meniscus of the solution at inlet *i*, and *r*_*i*_ is the radius of inlet *i* (see inset of Fig. 1a). For eqn (1), the shape of inlet meniscus is assumed to be a spherical cap. This is because gravitational forces on the inlet meniscus are negligible with the small Bond number, Bo = *ρgL*^2^/*σ* < 0.02. Here, *L* is the characteristic length and we used the meniscus height. Also, each inlet meniscus is assumed to be pinned to the rim of the inlet. We experimentally confirmed the meniscus pinning at the inlet rim under the condition of height-to-radius ratio *h*_*i*_/*r*_i_ < 0.5 and solution contact angle > 49° (Section 3 of ESI†). We note that the condition *h*_*i*_/*r*_*i*_ < 0.5 is satisfied in normal surface tension-driven devices. Also, the condition of solution contact angle > 49° is normally met, because the inlets of surface tension-driven devices typically use hydrophobic or moderately hydrophilic surfaces to confine the solution menisci to the rims of their inlets. Thus, our condition for meniscus pinning at the inlet rim can be considered as valid in most cases.

The pressure (*P*_JCT_) at the junction of the channels is obtained by the analogy of Kirchhoff's current law and is given by2
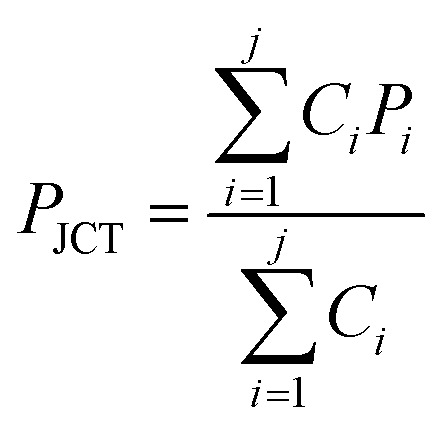
where *C*_*i*_ is the fluidic conductance of channel *i*, which is the inverse of channel fluidic resistance. The change rate of *h*_*i*_ is given by3
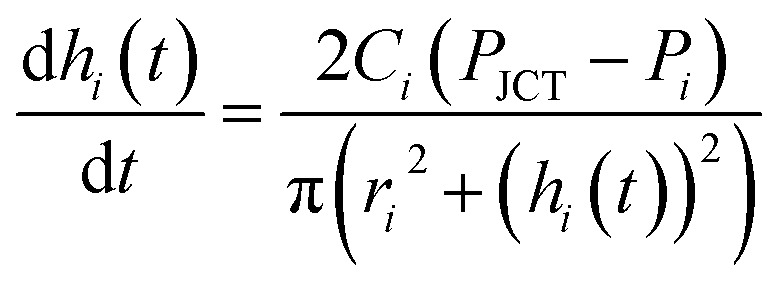


Eqn (3) is obtained by the condition where change rate of inlet volume is the same with the flow rate in the channel that connects the corresponding inlet and the junction. In addition, for eqn (2) and (3) we use Poiseuille's law, which neglects the contribution of flow inertia in the relation between pressure and flow rate. This is because the Reynolds number is small (Re < 0.5) in our system. In eqn (3), the number of *h*_*i*_ increases with increasing step number. For example, in step 2, *h*_1_ and *h*_2_ are used; and in step 3, *h*_1_, *h*_2_, and *h*_3_ are used. The corresponding numbers of *P*_*i*_, *r*_*i*_, and *C*_*i*_ also increase with increasing step number. To solve the simultaneous differential form of eqn (3), we measured the initial values of *h*_*i*_ in each step, and used them as the initial condition of each step. The equations were numerically solved with MATLAB.

## 3. Experimental

We fabricated devices using soft lithography technique.^25^ The devices have two layers. The top layer was produced from hydrophobic polydimethylsiloxane (PDMS) with channel features, while the bottom layer was comprised of hydrophilic slide glass. The hydrophilicity of the slide glass was increased by an air plasma machine (Femto Science) so that the solutions injected at the inlets can spontaneously fill the channels and meet at the junction. The dimensions of inlets and channels were measured to calculate the fluidic conductance of each channel. The channel dimensions were in the range 60–100 μm (*h*) × 180–400 μm (*w*) × 1.4–50 mm (*l*), with inlet radii ranging from 1 to 2.5 mm. The channel dimensions varied *C*_*i*_ in the range of 7 × 10^−14^ to 9 × 10^−12^ m^5^ N^−1^ s^−1^. We used a stereo microscope (Olympus) and digital microscope (Dino-lite) to measure the meniscus height of each inlet. We used de-ionized water for the working solution and food dyes were added for flow visualization.

## 4. Results and discussion

### 4.1. Backflow formation process

We analyze backflow formation with our model. Fig. 1d and e show the theoretical change in pressures that correspond to cases 1 (strong backflow) and 2 (weak backflow) in Fig. 1b, respectively. The solutions move from high to low pressure positions. In step 3a (gray region in Fig. 1d and e), the pressure condition is *P*_3_ > *P*_JCT_ > *P*_2_ (*P*_I2_ = *P*_I1_). Thus, solution 3 at inlet 3 moves through the junction to inlets 1 and 2. Owing to the fluidic motions, *P*_3_ decreased and *P*_1_ and *P*_2_ increased. In step 3b (white region), *P*_2_ becomes greater than *P*_JCT_, which reverses the flow direction of solution 2 from inlet 2 to the junction. Thus, because *P*_2_ > *P*_JCT_ > *P*_1_, solution 2 moves through the junction toward channel 1 and inlet 1. We note that the backflow volume of solution 2 is different for the two cases. The insets of Fig. 1d and e present the volume ratio (*V*_2_/*V*_Ch2_) of the inlet meniscus volume (*V*_2_) and channel 2 volume (*V*_Ch2_). *V*_Ch2_ was fixed at 30 nL, and the initial value of *V*_2_ was expressed as 0 by subtracting its initial volume from *V*_2_. During backflow, *V*_2_/*V*_Ch2_ decreases because solution 2 is released from inlet 2. To quantify the backflow volume at inlet 2, we define normalized backflow volume (Δ*V*_B2_, see the insets) at inlet 2 as Δ*V*_B2_ = (*V*_2P_ − *V*_2E_)/*V*_Ch2_, where *V*_2P_ and *V*_2E_ are the volumes of inlet 2 meniscus at the peak and equilibrium states of *P*_2_, respectively. The corresponding backflow pressure at inlet 2 (Δ*P*_B2_) is defined in Fig. 1d. If Δ*V*_B2_ > 1 (*i.e.*, *V*_2P_ − *V*_2E_ > *V*_Ch2_), then the volume that goes out of inlet 2 is greater than the channel 2 volume, so the backflow of solution 2 moves through channel 2 to another channel. This case is shown in the inset of Fig. 1d, where Δ*V*_B2_ = 2.8 > 1 (case 1 in Fig. 1b). In contrast, in the inset of Fig. 1e, Δ*V*_B2_ = 0.6 < 1, so solution 2 does not go out of channel 2 even for its backflow (case 2 in Fig. 1b). We analyzed how *P*_I*i*_, *r*_*i*_, and *C*_*i*_ influence the backflow strength at inlet 2.

We note that the backflow shown in this system does not result from inertia of fluids. This is because the Reynolds number (Re), which is the ratio of inertia to viscous force, was Re < 0.5 in our system. For the inertia effect to be dominant, Re needs to be high. Indeed, the studies that reported backflows even in single channel systems had Re > 10, owing to large channel size and high inlet pressure driven by repeated injection of small drops.^26–29^ In addition, we used a solution containing food dye with the same concentration (3.6% by weight). Thus, there was no flow induced by concentration gradient.

### 4.2. Effect of fluidic conductance and channel size on the backflow

**Fig. 2:**
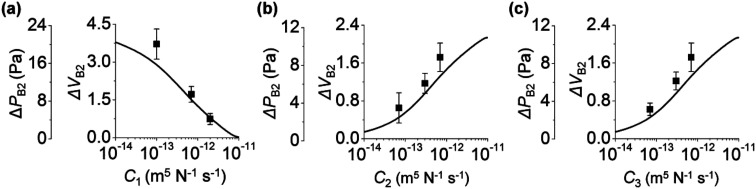
Influence of *C*_*i*_ on the backflow of inlet 2. When one fluidic conductance was changed, the others were fixed at 7 × 10^−12^ m^5^ N^−1^ s^−1^. Here, *r*_*i*_ = 2, 1, and 1 mm (*i* = 1 to 3). In step 3, *P*_I*i*_ = 35, 35, and 75 Pa. Variations of Δ*P*_B2_ and Δ*V*_B2_ by *C*_1_ are shown in (a), by *C*_2_ in (b), and by *C*_3_ in (c). Lines and points are the theoretical and experimental (*n* = 3) values, respectively.

We study the effect of the fluidic conductance of channel *i* (*C*_*i*_, *i* = 1 to 3) on the backflow. When one fluidic conductance was changed, the others were fixed at 7 × 10^−12^ m^5^ N^−1^ s^−1^. In step 3, inlet 3 releases solution 3 to the other inlets, inlet 2 is the place where backflow occurs, and inlet 1 takes the backflow solution. We analyze the backflow of inlet 2 under the change of *C*_*i*_. Fig. 2a shows that high *C*_1_ decreases Δ*P*_B2_ and Δ*V*_B2_. This behavior is explained by the fast rise of *P*_1_ and its resultant small difference between *P*_1_ and *P*_2_. When *C*_1_ increases, solution 3 injected from inlet 3 can easily move to inlet 1 rather than inlet 2. This is because comparatively high fluidic conductance of channel 1 allows rapid fluidic transport through channel 1 to inlet 1. As a result, *P*_1_ rapidly increases, according to the comparison *P*_1_ in the gray region of Fig. 1e and d. When *P*_1_ becomes high, the difference between *P*_2_ and *P*_1_ decreases (step 3b in Fig. 1d and e). Thus, the backflow that moves from inlet 2 toward inlet 1 decreases. Compared to *C*_1_, high *C*_2_ increases Δ*P*_B2_ and Δ*V*_B2_ (Fig. 2b). This is because, with high *C*_2_, solution 3 can easily move through channel 2 to inlet 2, thereby causing a more rapid increase in *P*_2_ in step 3a. Similar to *C*_2_, *C*_3_ increases Δ*P*_B2_ and Δ*V*_B2_ (Fig. 2c). With high *C*_3_, inlet 3 releases solution 3 more rapidly to the other channels through channel 3. This significantly increases *P*_2_ in step 3a and leads to high Δ*P*_B2_ and Δ*V*_B2_ in step 3b. In addition, when the size of channel 2 increases under constant condition of *C*_2_, Δ*V*_B2_ decreases. This result is analyzed in Section 4 of ESI.†

The backflow of inlet 2 by the change of *C*_*i*_ can be collectively explained. High *C*_2_ and *C*_3_ increase backflow of inlet 2. This is because, at step 3a, solution 3 moves more rapidly to inlet 2 through channels 2 and 3 owing to high *C*_2_ and *C*_3_. On the other hand, high *C*_1_ decreases the backflow of inlet 2. This is because solution 3 moves more to inlet 1 rather than inlet 2 by the high fluidic conductance of channel 1 in step 3a and finally reduces the backflow of inlet 2 by the small pressure difference between inlets 1 and 2.

### 4.3. Influence of inlet radius and inlet pressure on the backflow

**Fig. 3:**
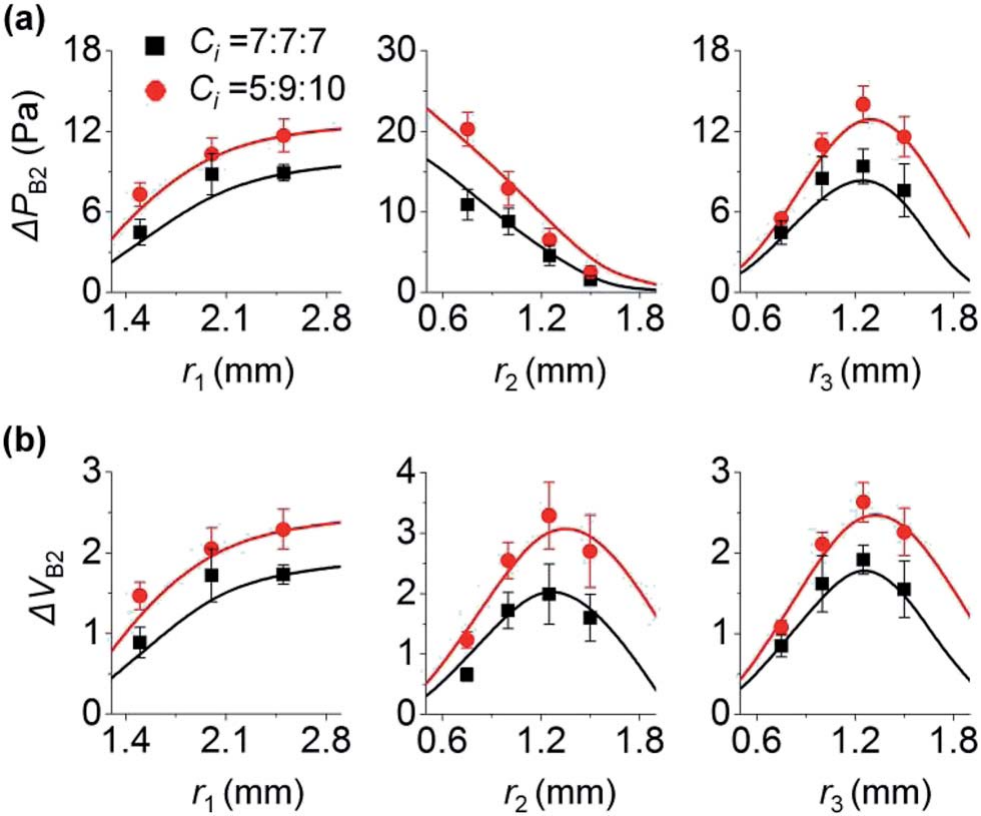
Influence of inlet radius *r*_*i*_ on the backflow of inlet 2. Unless otherwise noted, *r*_1_ = 2 mm, and *r*_2_ = *r*_3_ = 1 mm. In step 3, *P*_I*i*_ = 35, 35, and 75 Pa (*i* = 1 to 3). Δ*P*_B2_ and Δ*V*_B2_ are shown in (a) and (b), respectively. Lines and points are the theoretical and experimental (*n* = 3) values, respectively.

Here, we analyze the effect of inlet radius on the backflow of inlet 2. Fig. 3a shows how the three inlets influence Δ*P*_B2_. First, the left panel of Fig. 3a shows that high *r*_1_ increases Δ*P*_B2_. This is because high *r*_1_ makes the change of *P*_1_ gradual (eqn (1)), leading to larger difference between *P*_1_ and *P*_2_ at the end of step 3a (Fig. 1d). As a result, Δ*P*_B2_ increases in step 3b. For the two cases of *C*_*i*_ (7, 7, and 7; and 5, 9, and 10 (×10^−12^) m^5^ N^−1^ s^−1^), this trend is maintained. Second, the middle panel of Fig. 3a shows that high *r*_2_ decreases Δ*P*_B2_. This is because increasing *r*_2_ reduces the change rate of *P*_2_ (eqn (1)), and *P*_2_ gradually increases in step 3a (Fig. 1d). Thus, difference between *P*_1_ and *P*_2_ at the end of step 3a in Fig. 1d becomes small, thereby reducing Δ*P*_B2_. Third, unlike the cases of *r*_1_ and *r*_2_, increasing *r*_3_ does not monotonously vary Δ*P*_B2_ (right panel of Fig. 3a). When *r*_3_ increases from 0.6 to 1.2 mm, Δ*P*_B2_ increases. Herein, the initial pressure of inlet 3 was kept at *P*_I3_ = 75 Pa regardless of *r*_3_. To meet the constant *P*_I3_ condition even for increasing *r*_3_, the initial meniscus volume of inlet 3 increases (Section 5 of ESI†). Consequently, a larger amount of solution 3 goes from inlet 3 to inlet 2, thereby increasing Δ*P*_B2_. On the other hand, when *r*_3_ becomes greater than 1.2 mm, Δ*P*_B2_ decreases. We explain this result by using the difference between *P*_2_ and *P*_JCT_ that influences the flow from inlet 2 to the junction. Backflow occurs in step 3b only when *P*_2_ > *P*_JCT_ (Fig. 1d). Importantly, backflow decreases if *P*_2_ − *P*_JCT_ is small in step 3b; compare Fig. 1d and e. High *r*_3_ let *P*_JCT_ stay at a high value through *P*_3_ in step 3b (Fig. 1d). This is because *P*_JCT_ is considered as the weighted average of inlet pressures including *P*_3_ (eqn (2)) and slow decrease of *P*_3_ by high *r*_3_ let *P*_JCT_ remain at a high value. Accordingly, pressure difference between *P*_JCT_ and *P*_2_ decreases in step 3b, decreasing Δ*P*_B2_.

Collectively, when inlet 1 receives backflow from inlet 2, high *r*_1_ decreases the difference between *P*_1_ and *P*_2_ at step 3a, leading to lower Δ*P*_B2_ at step 3b. Oppositely, high *r*_2_ increases the difference between *P*_1_ and *P*_2_, thus increasing Δ*P*_B2_ at step 3b. Increasing *r*_3_ rises Δ*P*_B2_ at first because of large volume of inlet 3 meniscus, and then reduces decreases Δ*P*_B2_ because of the low difference between *P*_2_ and *P*_JCT_ at step 3b.

The change in Δ*V*_B2_ follows the trend of Δ*P*_B2_ when *r*_1_ and *r*_3_ vary (left and right panels of Fig. 3). As shown in Fig. 2, with *C*_*i*_ variation, Δ*V*_B2_ and Δ*P*_B2_ changed in the same manner. On the other hand, Δ*V*_B2_ varied differently with Δ*P*_B2_ with the change in *r*_2_ (middle panels of Fig. 3). Δ*V*_B2_ increases when *r*_2_ increases from 0.6 to 1.2 mm, and then decreases when *r*_2_ > 1.2 mm. The increasing *r*_2_ lets inlet 2 take more fluids from inlet 3 in step 3a (Section 6 of ESI†). This allows inlet 2 to release a greater amount of backflow volume in step 3b for 0.6 < *r*_2_ < 1.2 mm. However, with increasing *r*_2_, the reduction of *P*_2_ becomes more gradual in step 3b while *P*_JCT_ insignificantly changes by *r*_2_. This leads to the reduced difference between *P*_JCT_ and *P*_2_, thus decreasing Δ*V*_B2_ for *r*_2_ > 1.2 mm in step 3b.

**Fig. 4:**
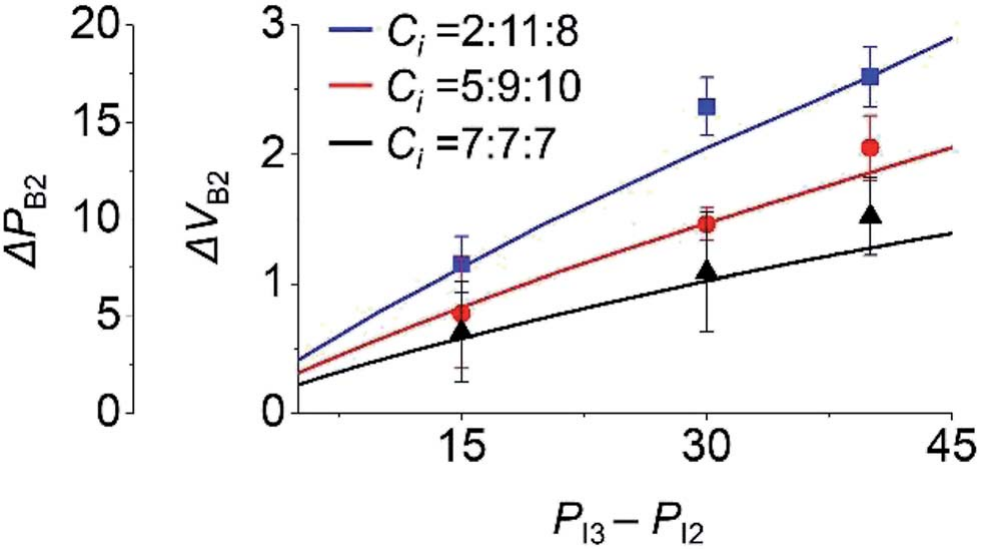
Effect of initial pressure (*P*_I*i*_) of inlet *i* in step 3 on the backflow of inlet 2. Lines and points are the theoretical and experimental (*n* = 3) values, respectively.

To study the effect of the initial inlet pressures (*P*_I*i*_) in step 3 on the backflow, *P*_I*i*_ values were varied and the backflow at inlet 2 was measured. Here, *P*_I3_ was 75 Pa for different values of *P*_I2_, with *P*_I2_ = *P*_I1_. We used three sets of *C*_*i*_, as shown in Fig. 4. Under this condition, Δ*P*_B2_ and Δ*V*_B2_ unanimously increased with the increasing *P*_I3_ − *P*_I2_. This is because at higher *P*_I3_ − *P*_I2_, a comparatively larger amount of inlet meniscus volume is used for inlet 3, which results in higher Δ*P*_B2_ and Δ*V*_B2_.

### 4.4. The effect of increasing step on reducing the backflow

**Fig. 5:**
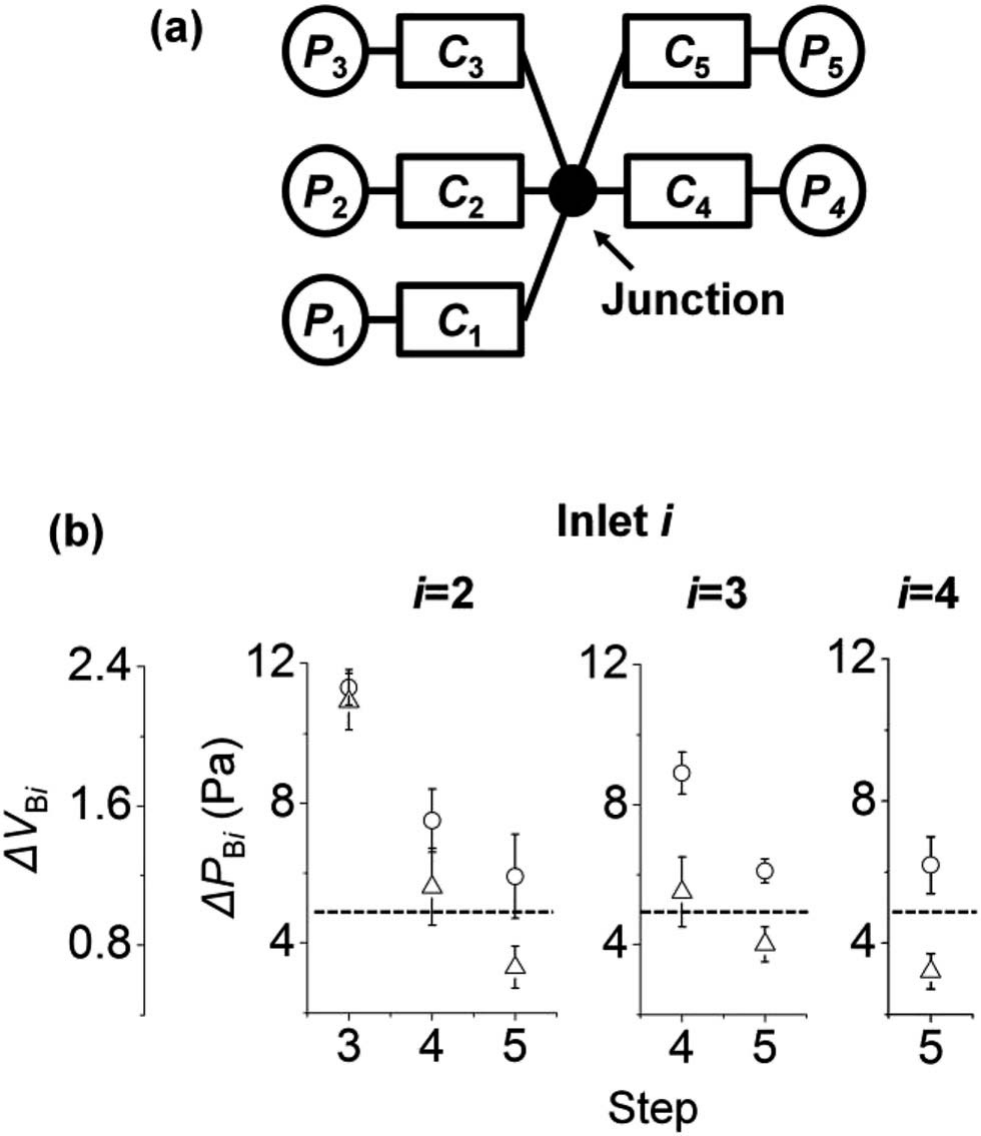
Backflow in surface tension-driven networks with five inlets and five channels. (a) Circuit diagram of the surface tension-driven network. (b) Change of Δ*P*_B*i*_ and Δ*V*_B*i*_ at inlet *i* (*i* = 2 to 4). Symbols designate subsets: circles for case 1; triangles for case 2. In case 1, *C*_*j*_ = 5, 10, 9, 13, and 13 (×10^−12^) m^5^ N^−1^ s^−1^ (*j* = 1 to 5). In case 2, *C*_*j*_ = 5, 10, 9, 5, 4 (×10^−12^) m^5^ N^−1^ s^−1^. For both cases, *r*_*j*_ = 2, 1, 1, 1, and 1 mm. For Δ*V*_B*i*_ < 1 (under the dashed line), the backflow of inlet *i* stays in channel *i* and does not go into other channels. The error bars were obtained from five experiments.

So far, we have studied the backflow in surface tension-driven networks with three inlets and three channels. Now, we expand our understanding to surface tension-driven networks with five inlets and five channels with one junction. Fig. 5a shows the circuit diagram of the surface tension-driven network. Solution *i* was injected at inlet *i* in step *i*, and the initial pressures of inlet *i* in step *i* were 35, 35, 75, 75, and 75 Pa, respectively. The left to right panels of Fig. 5b show the change in Δ*P*_B*i*_ and Δ*V*_B*i*_ at inlet *i* (*i* = 1 to 3). For example, inlet 4 (right panel) shows the changes in Δ*P*_B4_ and Δ*V*_B4_ only in step 5 without steps 3 and 4. This is because the injection to inlet 4 occurs in step 4, and inlet 4 is empty before step 4. Similarly, inlet 3 (middle panel) shows the variations in Δ*P*_B3_ and Δ*V*_B3_ only in steps 4 and 5, without step 3.

For inlets 2 and 3, Δ*P*_B*i*_ and Δ*V*_B*i*_ decrease as the step number increases. The initial pressures of inlets increase with increasing step number. This is because by adding solutions to inlets, the volumes of inlet menisci increase as the step number increases. On the other hand, we set the initial pressure of inlet *i* in step *i* (*i* = 3 to 5) as constant at 75 Pa. As a result, the initial pressure difference between inlet *i* and the other inlets in step *i* decreased. In Fig. 4, we explained that the backflow pressure at inlet 2 decreases if the pressure difference between *P*_I3_ and *P*_I2_ is reduced in step 3. Thus, similar to the case in Fig. 4, Δ*P*_B*i*_ and Δ*V*_B*i*_ decrease with increasing step number. In addition, if Δ*V*_B*i*_ is under the dashed lines (Δ*V*_B*i*_ < 1) in Fig. 5b, then the backflow of inlet *i* stays in channel *i* and does not go into other channels.

In addition, our model is broadly applicable to analyze the backflow of the paper microfluidic devices as well as polymer devices. This is because, even for a paper device with porous layer, its inlet pressure can be modeled as variable pressure source by Laplace pressure and its channels can be modeled as fluidic conductor. Specifically, although paper channels follow Darcy's law and polymer channels obey Poiseuille's law, in the two laws flow rate (*Q*) is commonly proportional to pressure difference (Δ*P*). That is, *Q* = *C*Δ*P*, where *C* is fluidic conductance of a channel. *C* is *Ak*/(*μL*) in a paper channel and is π*r*^4^/(8*μL*) in a polymer channel with circular cross section. Here, *A* is cross sectional area of channel, *μ* is viscosity, *L* is filled channel length, *k* is permeability, *r* is channel radius. Thus, because of the similarity between the two channel types, our model is applicable to paper microfluidic devices.

## 5. Conclusion

Our main contributions for a surface tension-driven network, where the pressure difference of the inlet menisci drives fluidic motions, are as follows: (i) developing a detailed model that describes the backflow strength of the surface tension-driven network; (ii) analyzing the contributions of network parameters (*e.g.*, fluidic conductance of channels, inlet radius, and initial pressure of inlets) to the backflow strength; (iii) characterizing the nonlinear relation between backflow volume and backflow pressure by the change of inlet radius; and (iv) presenting the change in backflow pressure with increasing step numbers.

We showed that each network element, including channel and inlet, has distinct characteristics that affect the change in inlet pressure. A large inlet size makes the change in the corresponding inlet pressure slow. Low fluidic conductance of a channel also makes the pressure change of the inlet directly connected to the channel slow. These two characteristics result in the complex behaviors of inlet pressures when the network elements constitute a surface tension-driven network. Backflow pressure decreases with the decrease (increase) in the corresponding fluidic channel conductance (inlet size). On the other hand, backflow volume reaches its peak value in the middle of the radius change of the corresponding inlet. This is because the inlet pressure was strongly affected by the radius change, but other inlet pressures were insignificantly influenced. In addition, we showed that backflow strength decreases when the initial pressure difference between the inlets decreases. Finally, we showed that in networks consisting of five inlet-channel elements, backflow pressure decreases as the step number increases because the pressure difference between the inlet where the solution is injected and the other inlets decreases. Findings in this study provide useful guidelines for controlling backflow in surface tension driven networks. Further studies could be performed to analyze and prevent backflows in more complex microfluidic networks with multiple junctions.

## Conflicts of interest

There are no conflicts of interest to declare.

## Supplementary Material

RA-009-C8RA09756A-s001
