# Supplementary material for: Influence of surface tension-driven network parameters on backflow strength
Source: RSC Adv. 2019 Apr 2;9(18):10345–51. doi: 10.1039/c8ra09756a (PMC9062321; doi:10.1039/c8ra09756a)
Supplement: RA-009-C8RA09756A-s001 [file RA-009-C8RA09756A-s001.pdf]

This journal is © The Royal Society of Chemistry 2019

Electronic Supplementary Information

1. Modeling pressures of inlet meniscus and channel junction.

By Young–Laplace equation, the meniscus pressure (P_i) of inlet i is given as a function of the radius of curvature (R_i) and the surface tension (σ); see Figure S1a:

$$P_i = \frac{2\sigma}{R_i(t)} \tag{S1}$$

If the meniscus at inlet i does not exceed the edge of inlet i, by the Pythagorean theorem, R_i is a function of the height (h_i) of the meniscus and the radius (r_i) of inlet i:

$$R_i(t) = \frac{h_i(t)^2 + r_i^2}{2h_i(t)} \tag{S2}$$

Equation (S2) is obtained by the condition where inlet meniscus is pinned at the inlet rim. By inserting eq S2 into eq S1, we can rewrite P_i as

$$P_i = \frac{4\sigma h_i(t)}{(h_i(t))^2 + r_i^2} \tag{S3}$$

Then, the sum of flow rates passing through the channel junction is obtained by the analogy of Kirchhoff's current law (see Figure S1b):

$$\sum_{i=1}^{k} C_i(P_{\mathrm{JCT}} - P_i) = 0 \tag{S4}$$

where C_i is the fluidic conductance of channel i, which is the inverse of fluidic resistance; and P_{JCT} is the channel junction pressure. Herein, channel fluidic resistance, $R_i = a_i L_i$

$$a_i = \frac{3\mu_j}{64 w_i h^3}\left[1 - \frac{192h}{\pi^5 w_i}\sum_{n=1,2,3...}^{\infty}\frac{\tanh(\frac{(2n-1)\pi w_i}{2h})}{(2n-1)^5}\right]^{-1} \tag{S5}$$

where μ_{j} is dynamic viscosity of each solution that passes the microchannel, and w_{i} L_i, and h are the width, length, and height of the microchannels, respectively.

Then, the junction pressure P_{JCT} is

$$P_{\text{JCT}} = \frac{\sum_{i=1}^{k} P_i C_i}{\sum_{i=1}^{k} C_i} \tag{S6}$$

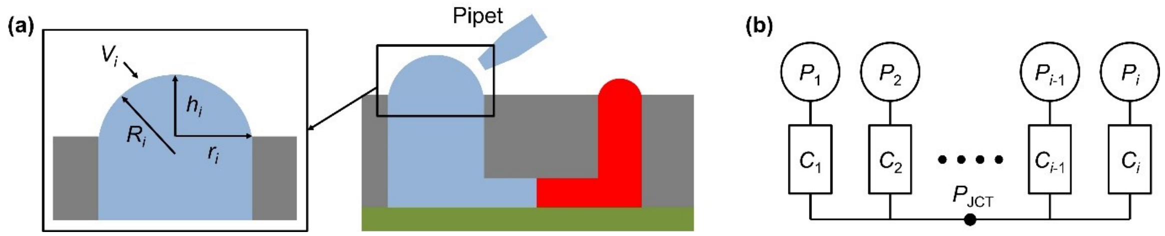

Figure S1. (a) Meniscus of inlet and generation of Laplace pressure. (b) Diagram showing fluidic circuit.

2. Modeling the relation between meniscus height and inlet pressure.

Owing to the similarity between fluidic resistance and electrical resistance, the flow rate (Q_i) from the channel junction to inlet *i* via channel *i* can be expressed as (see Figure S1b)

$$Q_i = C_i (P_{\text{JCT}} - P_i) \tag{S7}$$

In step *k,* the volume of the meniscus (V_i,) at inlet *i* is obtained by

$$V_i = \frac{\pi}{6} h_i (3r_i^2 + (h_i)^2) \tag{S8}$$

The change rate of V_i is thus expressed as

$$\frac{dV_i}{dt} = \frac{\pi(r_i^2 + (h_i(t))^2)}{2} \frac{dh_i(t)}{dt} \tag{S9}$$

Becuase dV_i/dt is equal to Q_i, by applying eq S6 into eq S8, we obtain

$$\frac{dh_i(t)}{dt} = \frac{2C_i(P_{\text{JCT}} - P_i)}{\pi(r_\text{i}^2 + (h_i(t))^2)} \tag{S10}$$

3. Meniscus pinning condition at the inlet rim

We experimentally confirmed the meniscus pinning at the inlet rim at the condition of (1) height-to-radius ratio $h_i/r_i < 0.5$ and (2) solution contact angle >49°. A stereo microscope was used to observe the side view of menisci at the inlet rims. To have different values of the water contact angles, the PDMS surface was plasma treated. By adjusting the plasma power and treatment duration, we obtained equilibrium contact angles of 49, 70, and 105° on the PDMS surface. By the constant flow input (0.5 μL/s) from a syringe pump, the inlet volume was increased and the inlet meniscus became more convex (Fig. S2a). The inlet meniscus remains pinned at the inlet rim up to 42 s. Then, the contact line of the meniscus moves over the rim. As such, we measured the maximum meniscus height (h_{max}) that maintains meniscus pinning for inlet radii (r) of 1 and 2 mm and contact angles of 49, 70, and 105°. Fig. S2b shows that the value of h_{max} / r increases with increasing contact angle.

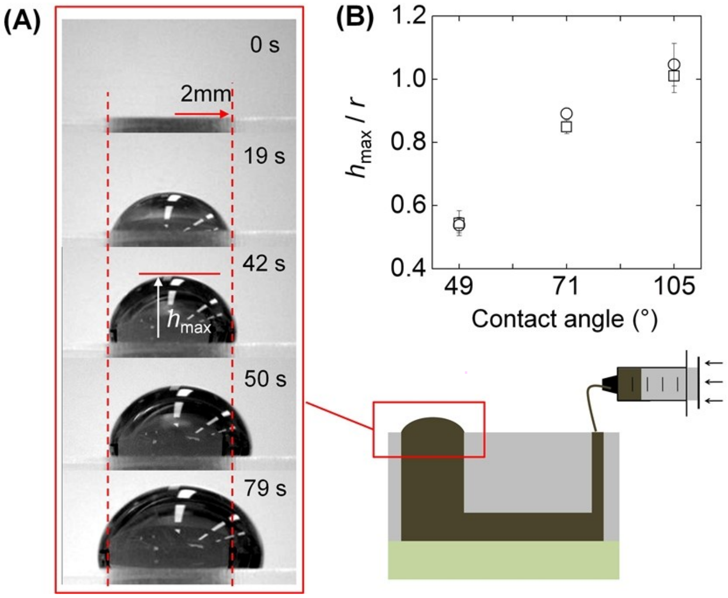

Fig. S2. Experimental validation of meniscus pinning at inlet rims. (A) Experimental setup and time lapse photos of the inlet meniscus. (B) Ratio of maximum meniscus height to inlet radius (h_{max} / r) that preserve the meniscus pinning condition under the change of the contact angle. Here, symbols of rectangle and circle correspond to r = 1 and 2 mm, respectively.

4. Change of meniscus height and volume of inlet by inlet radius

We analyzed the effect of the size of channel 2 on ΔV_{B2}, under constant condition of C_2. Fig. S3 shows that ΔV_{B2} decreases with the increase in the width (W) and height (h) of channel 2. When W and h increase, the length (L) of channel 2 increases to keep C_2 constant. Because $V_{Ch2} = WhL$, V_{Ch2} increases. However, V_2, which is the inlet meniscus volume, does not change because the other conditions do not change. Thus, ΔV_{B2} decreases because $\Delta V_{B2} = V_2/V_{Ch2}$.

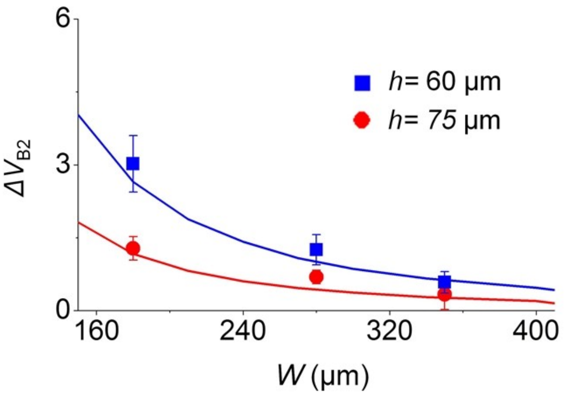

Fig. S3. Effect of the size of channel 2 on the normalized backflow volume (ΔV_{B2}). W and h are the width and height of channel 2, respectively. Lines and points are the theoretical and experimental (n = 3) values, respectively. Here, C_i = 5, 9, and 10 ($\times 10^{-12}$) m^5 N^{-1} s^{-1} (i = 1 to 3), and r_i = 2, 1, and 1.25 mm. In step 3, P_{Ii} = 35, 35, and 75 Pa.

5. Change of meniscus height and volume of inlet by inlet radius

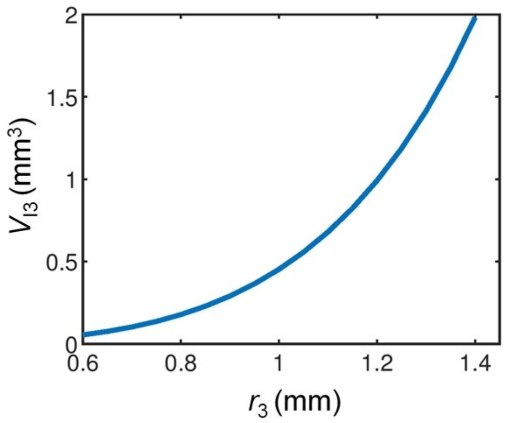

Fig. S4. Theoretical relation between r_3 and initial meniscus volume (V_{I3}) of inlet 3. The result is obtained by applying eq (S3) to (S7). P_3 is set as 75 Pa.

6. Change of volume ratio and inlet pressures by radius of inlet 2

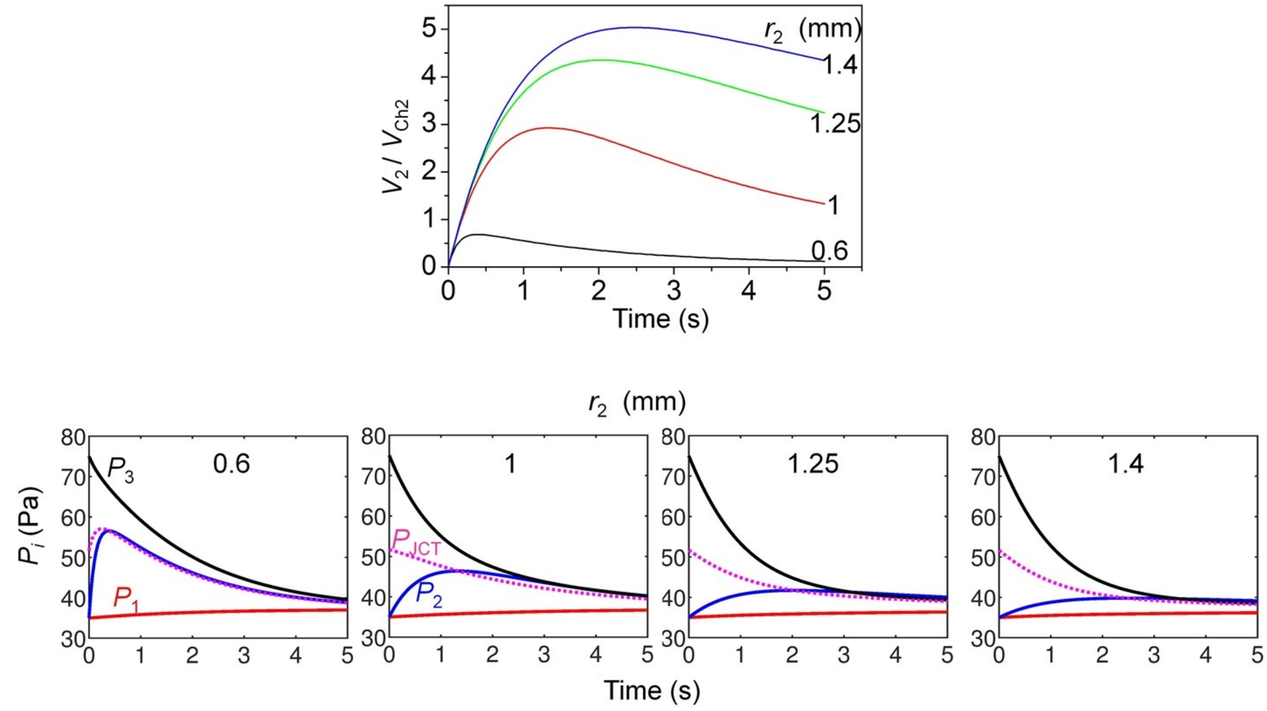

Fig. S5. Top graph shows the variation of the volume ratio (V_2/V_{Ch2}) of the inlet meniscus volume (V_2) and channel 2 volume (V_{Ch2}) in step 3. Bottom graphs show temporal change of inlet and junction pressures in step 3. In step 3, P_{Ii} values (i = 1 to 3) are 35, 35, and 75 Pa; r_1 and r_3 values are 2 and 1 mm; and C_i values are 5, 9, and 10 ($\times10^{-12}$) m^5 N^{-1} s^{-1}, respectively.
